# Detection of Aggregation-Competent Tau in Neuron-Derived Extracellular Vesicles

**DOI:** 10.3390/ijms19030663

**Published:** 2018-02-27

**Authors:** Francesc X. Guix, Grant T. Corbett, Diana J. Cha, Maja Mustapic, Wen Liu, David Mengel, Zhicheng Chen, Elena Aikawa, Tracy Young-Pearse, Dimitrios Kapogiannis, Dennis J. Selkoe, Dominic M. Walsh

**Affiliations:** 1 Laboratory for Neurodegenerative Disease Research, Ann Romney Center for Neurologic Diseases, Brigham & Women’s Hospital and Harvard Medical School, Boston, MA 02115, USA; trescomacatorze@gmail.com (F.X.G.); gtcorbett@bwh.harvard.edu (G.T.C.); dcha1@bwh.harvard.edu (D.J.C.); wliu@bwh.harvard.edu (W.L.); dmengel@partners.org (D.M.); zhicheng.chen@gmail.com (Z.C.); tyoung@rics.bwh.harvard.edu (T.Y.-P.); dselkoe@bwh.harvard.edu (D.J.S.); 2 Laboratory of Neurosciences, National Institute on Aging, NIH, Baltimore, MD 21224, USA; maja.mustapic@nih.gov (M.M.); kapogiannisd@mail.nih.gov (D.K.); 3 Center for Interdisciplinary Cardiovascular Sciences, Division of Cardiovascular Medicine, Brigham & Women’s Hospital and Harvard Medical School, Boston, MA 02115, USA; eaikawa@bwh.harvard.edu

**Keywords:** Alzheimer’s disease, biomarkers, cerebrospinal fluid, exosomes, iPSCs, neuron, plasma

## Abstract

Progressive cerebral accumulation of tau aggregates is a defining feature of Alzheimer’s disease (AD). A popular theory that seeks to explain the apparent spread of neurofibrillary tangle pathology proposes that aggregated tau is passed from neuron to neuron. Such a templated seeding process requires that the transferred tau contains the microtubule binding repeat domains that are necessary for aggregation. While it is not clear how a protein such as tau can move from cell to cell, previous reports have suggested that this may involve extracellular vesicles (EVs). Thus, measurement of tau in EVs may both provide insights on the molecular pathology of AD and facilitate biomarker development. Here, we report the use of sensitive immunoassays specific for full-length (FL) tau and mid-region tau, which we applied to analyze EVs from human induced pluripotent stem cell (iPSC)-derived neuron (iN) conditioned media, cerebrospinal fluid (CSF), and plasma. In each case, most tau was free-floating with a small component inside EVs. The majority of free-floating tau detected by the mid-region assay was not detected by our FL assays, indicating that most free-floating tau is truncated. Inside EVs, the mid-region assay also detected more tau than the FL assay, but the ratio of FL-positive to mid-region-positive tau was higher inside exosomes than in free solution. These studies demonstrate the presence of minute amounts of free-floating and exosome-contained FL tau in human biofluids. Given the potential for FL tau to aggregate, we conclude that further investigation of these pools of extracellular tau and how they change during disease is merited.

## 1. Introduction

Aggregation and deposition of the tau protein is a common feature of Alzheimer’s disease (AD) and a range of neurodegenerative disorders, collectively referred to as tauopathies [1,2]. In recent years, much attention has focused on the observation that histological deposits of tau appear to develop in a stereotypical spatial and temporal pattern linked to the appearance of disease symptoms [3]. This phenomenon has prompted many to suggest that tau is physically transferred from neuron to neuron along anatomical pathways [4,5,6]. This so-called ‘pathogenic spread model’ has at least four components: (1) release from donor neurons, (2) aggregation (which could occur before or after step 1), (3) uptake into certain recipient neurons, and (4) induction of aggregation in the recipient cells [7].

To date, the mechanisms underlying how tau is transferred between neurons, and the forms of tau released from neurons, remain unknown. Tau lacks a secretory signal and thus its release must occur by an unconventional mechanism. Possible release mechanisms include tunneling nanotubes, trans-synaptic transfer, and release of tau due to synaptic, neuritic, or neuronal compromise [7,8,9,10,11,12,13,14,15]. Regarding the potential for synaptic transfer, tau is not found in secretory vesicles and there are no compelling mechanisms to account for uptake into recipient cells. Similarly, it is not clear how ‘free-floating’ tau released by neuronal compromise could be taken up by a donor cell. Tunneling nanotubes, filamentous-actin-containing membranous structures that bridge and connect cells, have been found to contain tau [16], but the roles that tunneling nanotubes play in the brain are poorly understood. On the other hand, transcellular communication via extracellular vesicles (EVs), such as exosomes, is known to occur and offers a plausible mechanism by which tau may be released from one neuron and taken into another [17,18,19].

EVs consist of a lipid bilayer encapsulating cytosol and are released by all cells [20]. The loading of cargo (including proteins, DNA, and various forms of RNA) into EVs occurs in an apparently specific, but as of yet poorly, understood manner. The nature and origin of EVs are diverse and have been described based on: (i) size (microparticles, microvesicles (MVs), nanovesicles, or nanoparticles), (ii) cell or tissue of origin (prostasomes, oncosomes), (iii) proposed functions (calcifying matrix vesicles, argosomes, tolerosomes), and (iv) biogenesis and release pathways (ectosomes or exosomes) [21]. “Exosomes” is the term that is most frequently applied to EVs, but it is important to emphasize that exosomes are only a subset of EVs, and not all EVs are exosomes. Electron microscopy of actively shedding cells allowed EVs to be defined based on their site of biogenesis. Exosomes are formed by inward budding of multivesicular bodies (MVBs) to produce intraluminal vesicles, such that when MVBs fuse with the plasma membrane exosomes are released [22,23]. By contrast, MVs (which are also known as ectosomes) are shed directly from the plasma membrane [24,25,26]. Typically, exosomes appear as vesicles with diameters between 30–100 nm, whereas MVs are reported to span an overlapping but much broader size range of 50 to 1000 nm [21,25]. EVs larger than ~350–400 nm are thought to include plasma membrane fragments released during apoptosis, and, therefore, some have suggested it is more appropriate to describe MVs as ranging from ~50 to 400 nm [27]. Whatever the precise range of MVs, the size and composition of MVs and exosomes often overlap, and currently there are no means to completely separate or biochemically distinguish exosomes and MVs [28,29,30,31].

Previous studies have reported that certain forms of tau are present in EVs [13,32,33,34], and some investigators have suggested that tau contained in exosomes may facilitate aggregation of tau in recipient cells [33,35]. Since the microtubule binding repeat domains (MTBRs) of tau are required for its aggregation [36,37,38], we set out to determine if tau secreted in human neuronal exosomes contains MTBRs and is therefore aggregation-competent. For this purpose, we developed two sensitive assays specific for full-length (FL), MTBR-containing tau, which we applied to analyze exosomes from human iPSC-derived neuron (iN) conditioned media (CM), cerebrospinal fluid (CSF), and blood plasma. In each case, we detected small amounts of FL tau contained within exosomes, but this material accounted for <0.01% of free-floating mid-region tau. Within the free-floating pool, FL tau accounted for <1% of mid-region tau, but nonetheless was ~100-fold more abundant than the FL tau that is found in exosomes. These studies demonstrate the presence of free-floating and exosome-bearing FL tau in human biofluids and should prompt further investigation of these aggregation-competent pools of extracellular tau and how they change during disease.

## 2. Results

### 2.1. Human Ipscs-Derived Neurons Release Aggregation-Competent Tau in Exosomes

**Figure 1:**
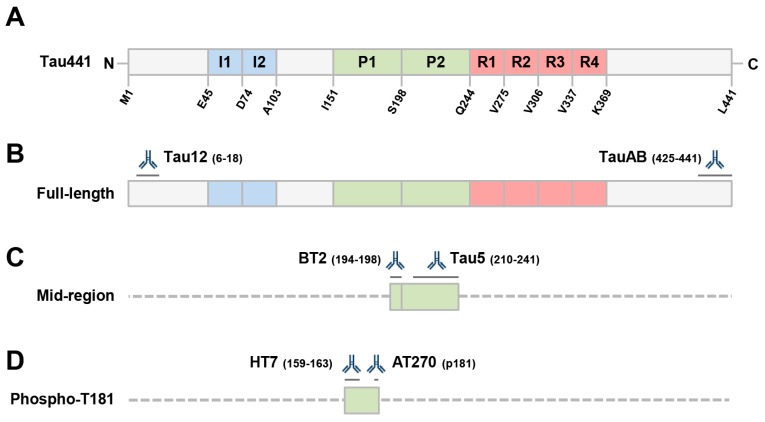
Schematic depicting the longest isoform of human tau (441), the antibodies employed and the tau fragment(s) recognized by immunoassays used in this study. (**A**) Domain structure of tau, including the 2 N-terminal inserts (I1 and I2), the proline-rich domain (P1 and P2), and the microtube-binding repeat domain (R1 to R4), which is required for tau aggregation. (**B**) Both our full-length ELISA and Simoa assays employ Tau12, a mAb to the extreme N-terminus of tau, and TauAB, a mAb to the extreme C-terminus. (**C**) Our mid-region assay employs mAbs BT2 and Tau5 which recognize epitopes in the proline-rich domain. This assay is highly similar to those used in clinical research. (**D**) The Innotest phospho-tau kit (from Fujirebio) employs 2 mid-region directed mAbs; HT7, which recognizes an epitope spanning residues 159–163, and AT270, a mAb specific for phosphorylated threonine 181. Epitopes for the antibodies are indicated by dark grey lines. Sequences of minimal predicted length recognized by a given ELISA are shown in filled boxes and other potentially detectable forms of tau are indicated with dashed lines.

We previously found that small amounts of tau are present in exosomes isolated from conditioned media (CM) of human and rodent neurons [13]. In those studies, we employed an immunoassay that was highly similar to clinically approved tests used to detect tau in human cerebrospinal fluid (CSF). Such assays, which employ the mid-region directed anti-tau mAbs BT2 and Tau5 (Figure 1), are often said to measure total tau. However, they are not expected to detect N- or C-terminal fragments that lack all or part of the mid-region. Furthermore, it is now appreciated that the majority of tau in CSF is composed of N-terminal and mid-region fragments [39,40,41,42], and that little full-length (FL) tau is released by healthy neurons [13]. Thus, a signal detected by mid-region assays cannot be assumed to reflect detection of FL tau (Figure 1). Here, we used novel assays specific for FL tau to explore whether FL (and therefore aggregation-competent) tau is present inside neuronal EVs of human origin.

**Figure 2:**
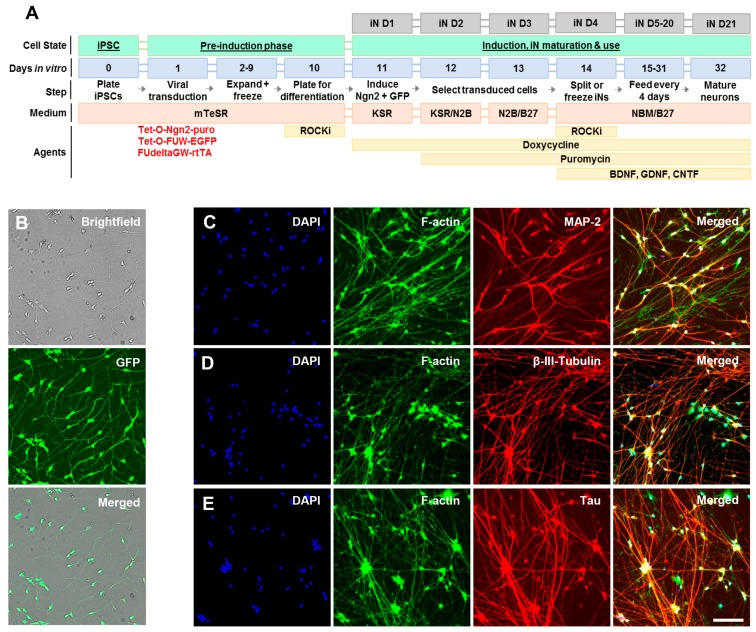
Neuronal differentiation of human iPSC lines. (**A**) Schematic depicting the procedure used to generate human induced neurons (iNs) from iPSCs. On day in vitro (DIV) 1, iPSCs were transduced with viruses expressing reverse tetracycline-controlled transactivator (rtTA), puromycin-resistant Neurogenin-2 (Ngn2-puro) and enhanced green fluorescent protein (EGFP). After expansion (DIV2–9), differentiation was induced with doxycycline on DIV11 (iN D1) and cells were matured for at least 23 days prior to harvesting conditioned medium (iN D23–33). (**B**) At iN D21, live cells were imaged using brightfield and FITC filters to confirm differentiation and selection. Cultures were then fixed and immunostained for DAPI, F-actin, and the neuron-specific proteins MAP-2 (**C**), β-III-Tubulin (**D**), and tau (**E**). Scale bar: 100 µm.

**Figure 3:**
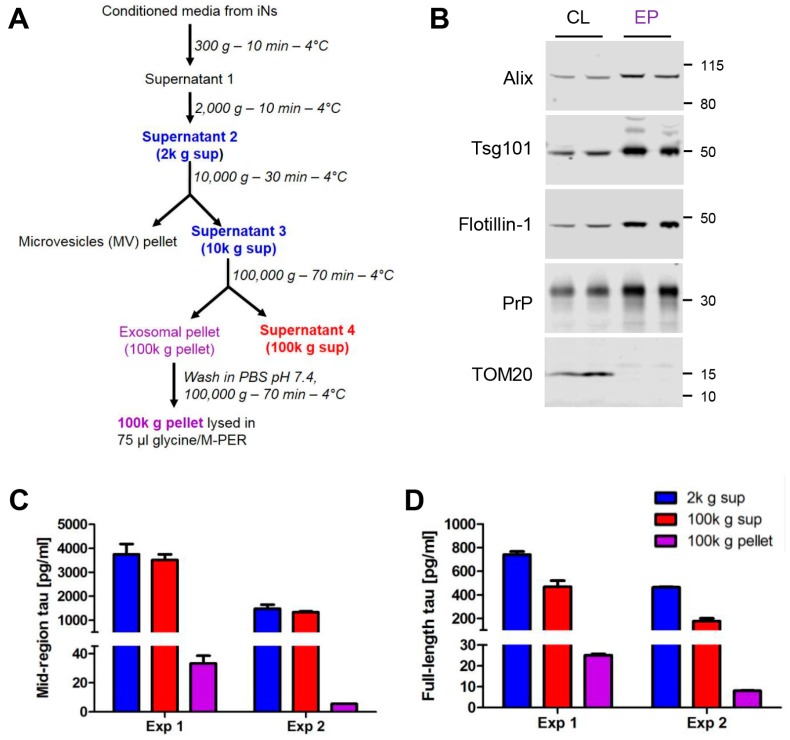
Exosomes from human iPSC-derived neurons contain detectable levels of full-length, aggregation-competent tau. (**A**) Flow chart describing the centrifugation procedure used to isolate exosomes from conditioned medium (CM). The speed, duration and temperature of each centrifugation step are indicated to the right of the arrows. Immediately after collection, CM was centrifuged at 300× *g* to remove dead cells and then 2000 g to remove cellular debris. The clarified CM was then centrifuged at 10,000× *g* to pellet microvesicles. The resulting supernatant (S3) was centrifuged at 100,000× *g* for 70 min to pellet exosomes, and these were washed once in PBS. The wash supernatant was discarded and the final exosomal pellet was lysed in glycine/MPER buffer. CM from two separate iN cultures, designated Experiment (Exp) 1 and Exp 2, was collected and processed as described. A portion of exosomal pellet (EP) lysate was analyzed by Western blotting alongside thecell lysates (CL) of iNs from which the exosomes were derived (**B**). Antibodies used are indicated on the left and the migration of molecular weight standards (in kDa) is on the right. Tau was measured in CM (2000 g (blue) and 100,000 g (red) supernatants and the lysed 100,000 g exosome pellet (purple) using our mid-region (**C**) and full-length (FL) (**D**) ELISAs. Concentrations represent the amount of analyte present in 1 mL of starting iN CM and account for dilution and concentration steps. Results for each culture (Exp 1 and 2) represent mean ± SD of technical triplicates.

Initial experiments focused on CM from iPSC-derived neurons. Human neurons were generated from a control iPSC line [43] using a modified version of the Neurogenin-2 differentiation protocol [44], as described in the Materials and Methods and illustrated in Figure 2A. iNs are mature by iN day 14 [45] and express neuronal markers including MAP-2, β-III tubulin and tau (Figure 2B–E). Media were collected from mature iN cultures between iN days 23–33 and centrifuged at progressively higher speeds to remove free-floating cells, cellular debris, and microvesicles. Small EVs (commonly referred to as exosomes) were harvested at 100,000 g (Figure 3A) [46] and analyzed alongside cell lysates by Western blotting for four proteins known to be enriched in exosomes and the mitochondrial outer membrane marker TOM20, which is not found in exosomes (Figure 3B). The supernatants from the 2000 g, and 100,000 g spins, plus lysates of the 100,000 g pellets were then analyzed for tau using our mid-region (Figure 3C) and FL assays (Figure 3D). Although the absolute levels of tau detected in iN, CM, and EV lysates varied between iN cultures prepared and conditioned on different days, the relative levels of mid-region-to-FL tau, and free-floating-to-exosomal tau were highly similar. CM supernatants contained approximately five times more tau detected by the mid-region assay than by the FL assay. These data are consistent with previous studies in which we used primary rat hippocampal neurons and found that FL tau contributed less than 20% of the signal detected by the mid-region assay [13]. Also, in accord with our prior analysis of rat neurons, the 2,000 g and 100,000 g supernatants contained much higher levels of tau than was present in the exosome pellets. However, as we observed with neuronal lysates [13], the FL and mid-region assays detected comparable amounts of tau in exosome lysates (Figure 3C,D). For instance, in the exosome pellet from Experiment 1, the mid-region and FL tau assays detected ~33 pg/mL and ~25 pg/mL tau, respectively; and, ~6 pg/mL and ~8 pg/mL tau in the exosome pellet from Experiment 2 (Figure 3C,D). It is important to note that the same recombinant tau standard was used for both the FL and mid-region ELISAs, making the concentrations that were measured comparable. These data suggest that almost 80% of the tau present in iN exosomes is full-length, and therefore competent to self-aggregate and potentially seed aggregation upon entry into recipient cells.

### 2.2. Full-Length Tau Is Present in CSF at Low Levels Both Free-Floating and Inside Exosomes

**Figure 4:**
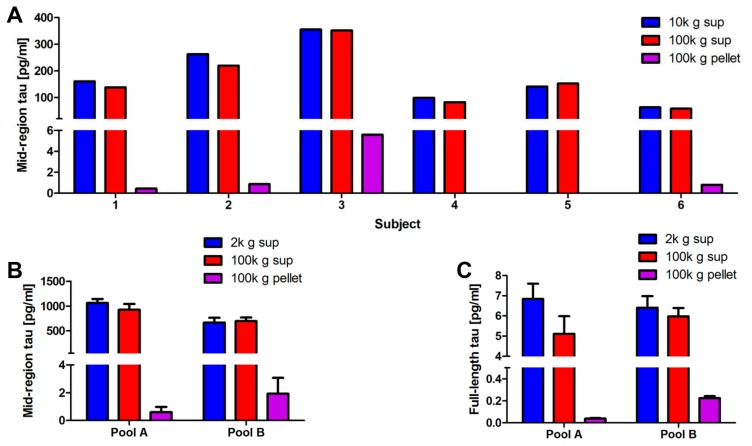
Exosomes from human CSF contain detectable amounts of full-length, aggregation-competent tau. (**A**) CSF samples from six healthy controls were processed by serial centrifugation, as described in Figure 3A. Free tau was measured in CSF in 10,000 g supernatant (blue) 100,000 g supernatant (red), and the 100,000 g pellet (purple). The majority of tau is free-floating, while a small amount of tau is recovered in the exosomal pellet of 4 out of 6 samples. Values represent the mean of technical replicates. (**B**,**C**) Two pools of CSF, designated Pool A and Pool B, were processed as above and the 2,000 g supernatants (blue) 100,000 g supernatants (red), and 100,000 g pellets (purple) analyzed using our mid-region ELISA (**B**) and FL Simoa assay (**C**). Concentrations represent the amount of analyte present in 1 mL of starting cerebrospinal fluid (CSF) and are corrected to account for dilutions incurred during the various processing steps. Results in (**A**) represent the mean of two technical replicates, and results in (**B**,**C**) indicate the mean ± SD of technical replicates of two independent experiments.

Having detected FL tau in iN CM, we searched for tau in CSF samples from six healthy controls using both our mid-region and FL ELISAs (Figure 4A). In accord with previous studies that documented that the majority of tau in CSF is C-terminally truncated [39,40,41,42], we detected tau in the 10,000 g and 100,000 g supernatant by the mid-region assay, but not the FL assay.

Values detected in CSF supernatants by the mid-region assay averaged around 200 pg/mL, and all of the values fell within the normal range expected for neurologically healthy individuals [47]. Importantly, in four of the six samples, small amounts of tau were detected inside exosomes by our mid- region assay. However, the levels were very low (0.44–5.6 pg/mL), with three samples yielding values close to the lower limit of quantification (LLoQ) of the assay, which, on the day of testing, was 30 pg/mL (i.e., equivalent to 0.44 pg/mL of tau in an exosomal lysate). Three of the samples that yielded detectable levels of tau in exosomal lysates had been processed on the same day and never frozen (subjects 1–3), whereas only one of the three samples (subjects 4–6) that had been frozen prior to exosome isolation yielded a detectable tau signal (Figure 4A). Due to the small number of samples examined, the low levels of tau detected in exosomes, and the inherent variability of biological specimens, it is not possible to determine whether freeze-thawing affects the recovery of exosomes from CSF. Given that prior studies have found freeze/thawing to have little effect on the recovery of EVs from blood [48], the apparent low recovery of tau from two frozen samples may simply reflect naturally lower levels of tau in exosomes from these particular samples.

Mindful of the low and variable levels of tau present in CSF exosomes, we developed an ultrasensitive Simoa-based immunoassay to search for FL tau. Simoa (named for single molecule array) technology is based upon the isolation of individual immunocomplexes on magnetic beads using standard ELISA reagents [49]. The main difference between Simoa and conventional immunoassays results from the use of an array containing 200,000 microwells, each of which is just large enough to accommodate one bead. The volume of the femtoliter-sized wells is two billion times smaller than traditional microtiter plate assays, so a single target molecule in a sealed microwell quickly generates enough fluorescence that can be readily measured. Positive wells are then enumerated, and the concentration of analyte in sample wells determined by reference to values obtained using known amounts of analyte. We used this ultra-sensitive assay to search for FL tau in 2 separate pools of human CSF (Figure 4B,C).

As with individual CSF samples (Figure 4A), tau was readily detected by the mid-region ELISA in the 2000 g and 100,000 g supernatants of the pooled CSF samples (Figure 4B), although the levels tended to be higher (1064 pg/mL in Pool A and 667 pg/mL in Pool B) in these pools than in the CSF from individual healthy controls (compare Figure 4A,B). Since the pooled samples were obtained from multiple human donors and no information is available about their clinical status, the modestly higher readings could be driven by one or more contributing donors who had AD or other conditions that are associated with elevated CSF tau. Importantly, tau was readily detected by our mid-region assay in the exosomal pellets from both CSF pool A and B. On the day of testing, the LLoQ of the mid-region ELISA was 7.81 pg/mL (i.e., equivalent to 0.12 pg/mL of tau in an exosomal lysate). Strikingly, small amounts of FL tau were also detected in the supernatants of CSF pools A and B and in the lysates of their 100,000 g exosomal pellets (Figure 4C). On the day of testing, the LLoQ for the FL Simoa assay was 0.74 pg/mL (i.e., equivalent to 0.01 pg/mL FL tau in an exosomal lysate), and the levels of tau in CSF exosomes were thus well within the reliable range of this assay. Detection of FL tau in CSF has been controversial [49], but our results indicate that small amounts of FL tau are present in at least some of the CSF samples. In the two CSF pools examined, FL tau accounted for only ~0.6–1% of the free-floating tau signal detected by our mid-region assay, and the maximum observed value for FL tau was ~7 pg/mL. Given these results, it is unsurprising that FL tau cannot be reliably detected in CSF using Western blotting or traditional ELISA methods.

As with iN exosomes (Figure 3), CSF exosomes contained tau detectable with both our mid-region ELISA and FL Simoa assay (Figure 4B,C). However, in CSF exosomes, the tau detected by the FL Simoa assay accounted for only ~6–12% of the tau detected by the mid-region ELISA (Figure 4A,B)—a much lower relative amount than in iN exosomes (Figure 3C,D). Nonetheless, the ratio of FL to mid-region tau was higher in exosomes (>6%) than exosome-depleted CSF (<1%; ([tau_FL_]/[tau_FL_ + tau_MR_]) (Figure 4B,C).

### 2.3. Optimization of the Method Used to Isolate Neuronal Exosomes from Plasma

**Figure 5:**
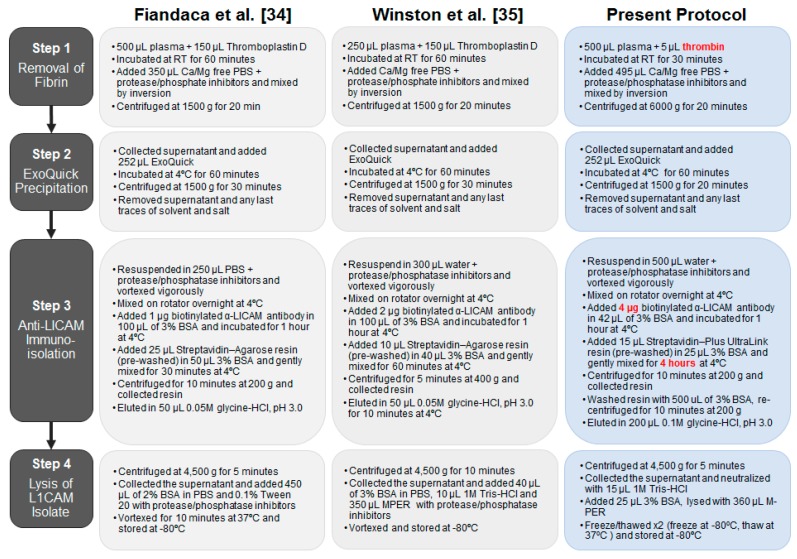
Comparison of published protocols used to isolate neuronal exosomes from blood and the method implemented in the present study. Preliminary studies revealed 2 steps (see red text) which critically influenced the production and analysis of neuronal exosomes: (i) the use of recombinant thrombin instead of thromboplastin D, and (ii) an increased amount of biotinylated anti-L1CAM and longer incubation time with streptavidin resin.

Having detected FL tau in CSF-derived exosomes, we next searched for tau in blood-borne, neuron-derived exosomes. The most widely used method for isolating neuronal exosomes from blood involves depleting plasma of clotting factors, precipitating EVs using ExoQuick, and immunoprecipitating neuronal exosomes using an anti-L1CAM antibody (Figure 5) [34,35,50,51].

Before analyzing precious clinical samples, we tested a number of modifications designed to further optimize this protocol. In the Goetzl/Kapogiannis protocol, clot formation was induced by the addition of thromboplastin D derived from rabbit brain [34], but we were concerned that the thromboplastin D might be contaminated with rabbit brain tau [52]. Thus, we compared the ability of thromboplastin D and recombinant thrombin to induce clot formation and tested whether they contained any exogenous tau. Both of the enzymes induced coagulation, but thrombin produced clots of more consistent size (Figure S1A). Moreover, when thromboplastin D was added to PBS and the solution processed as if it were plasma, a strong signal was detected by the ptau-181 (p181) and mid-region tau assays. In contrast, when thrombin was added to PBS and processed, no signal was detected by the p181 or mid-region ELISAs (Figure S1B). These results are in agreement with a very 

recent report [53] and indicate that the use of thrombin is preferable both because it allows for more consistent clot formation, and, most importantly, because it avoids contaminating samples with exogenous tau. In separate experiments, we also optimized the incubation time and amount of streptavidin-coated resin necessary to capture the biotinylated L1CAM antibody used to immunoisolate neuronal exosomes (Figure 5 Step 3, and Figure S2A–C).

With the optimized protocol, we proceeded to isolate exosomes from plasma samples obtained from a small cohort of clinically well-characterized human donors (Table 1). Neuronal exosomes were eluted from streptavidin resin, as outlined in Step 3 of our protocol (Figure 5). The major portion of the resulting exosome suspension from Step 3 (195 µL) was lysed (as described in Step 4), and a small portion of eluate (5 µL) was used without lysis to estimate the size distribution of vesicles using Nanoparticle Tracking Analysis (NanoSight, Amesbury, UK). Exosomal lysates were analyzed using two immunoassays for exosomes markers (a CD81 ELISA and a Tsg101 MSD immunoassay) and three different tau immunoassays (mid-region ELISA, FL tau Simoa, and Innotest p181 ELISA). Standards for each assay were prepared in the same buffer in which exosomes were lysed and buffers were confirmed to be compatible with each assay (Figure S3A–D, and Figure S4). Nanosight analysis of all 40 preparations (from Step 3) revealed a light scattering pattern indicative of EVs with diameters spanning ~65—305 nm (Table 2, Figure S5), and lysates (from Step 4) were also positive for the exosomal marker CD81 (Figure S6). Thus, the optimized method allows for the isolation of exosomes similar to those produced by the original Goetzl/Kapogiannis methodology [34,54], but which are free from extraneous sources of tau arising from the use of thromboplastin D.

### 2.4. Blood-Derived Neuronal Exosomes Contain Small Amounts of Aggregation-Competent Tau

**Figure 6:**
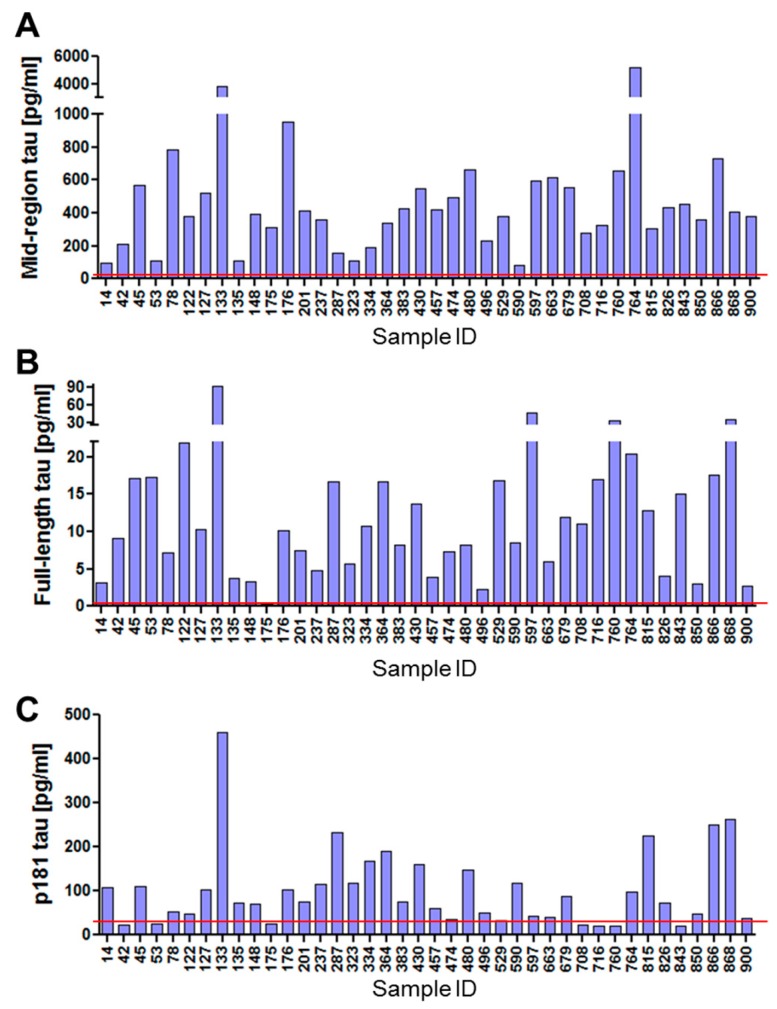
Blood-derived neuronal exosomes contain several different forms of tau. (**A**) Our in-house BT2-Tau5 ELISA detected tau in all plasma neuron-derived exosomes studied. On the day of testing the LLoQ for the assay was 7.81 pg/mL. (**B**) Full-length tau was detected with an in-house Simoa-based assay which on the day of testing had an LLoQ of 0.74 pg/mL. **(C)** The concentration of ptau-181 was measured with the Innotest commercial kit, which, on the day of testing, had an LLoQ of 24.5 pg/mL. For each assay, the LLoQ is indicated with a red line.

Previous studies reported detection of tau in neuronally-derived exosomes isolated from plasma [34,35,50,52,53,55,56,57,58]. However, several of those studies used thromboplastin D, which may have contaminated samples with exogenous tau [34,35]. More importantly, even if the tau detected did originate from human exosomes, the assays employed were directed to mid-region epitopes and thus it is not known whether any of the tau detected was full-length. Here, we show that the neuronally-derived exosomes analyzed in this study contained tau species detectable by at least two distinct immunoassays (Figure 6 and Table 2).

As seen with iN and CSF exosomes, the highest tau signal in neuron-derived exosomes isolated from plasma was detected with our mid-region assay (Figure 6 and Table 2). All 40 samples had mid-region signals comfortably above the LLoQ of the assay (7.81 pg/mL, i.e., equivalent to 0.12 pg/mL of tau in an exosomal lysate) ranging from ~91–5,200 pg/mL. The average concentration of mid-region tau in neuronal exosomes from plasma across all of the samples analyzed was 608.5 ± 149.2 pg/mL, while the concentration of FL tau was much lower (14.0 ± 2.5 pg/mL), such that FL tau accounted for only ~2% of the signal detected by the mid-region assay. These data indicate that, while a small fraction of tau in plasma neuronally-derived exosomes is full-length and therefore capable of aggregating, the vast majority is truncated. Certain prior studies detected low levels of p181-tau in plasma neuronally-derived exosomes [34,35,54], and here we also detected p181-tau in the majority (32 out of 40) of samples. There was an average of 100.4 ± 14.1 pg/mL p181-tau in the samples analyzed in this study, indicating that p181-tau accounted for less than 1/6th of the signal detected by mid-region tau. Collectively, these data present compelling evidence that multiple forms of tau are present in neuron-derived exosomes isolated from human plasma.

### 2.5. Levels of Different Types of Tau in Neuronal Exosomes from Healthy Controls, AD and MCI Subjects

**Figure 7:**
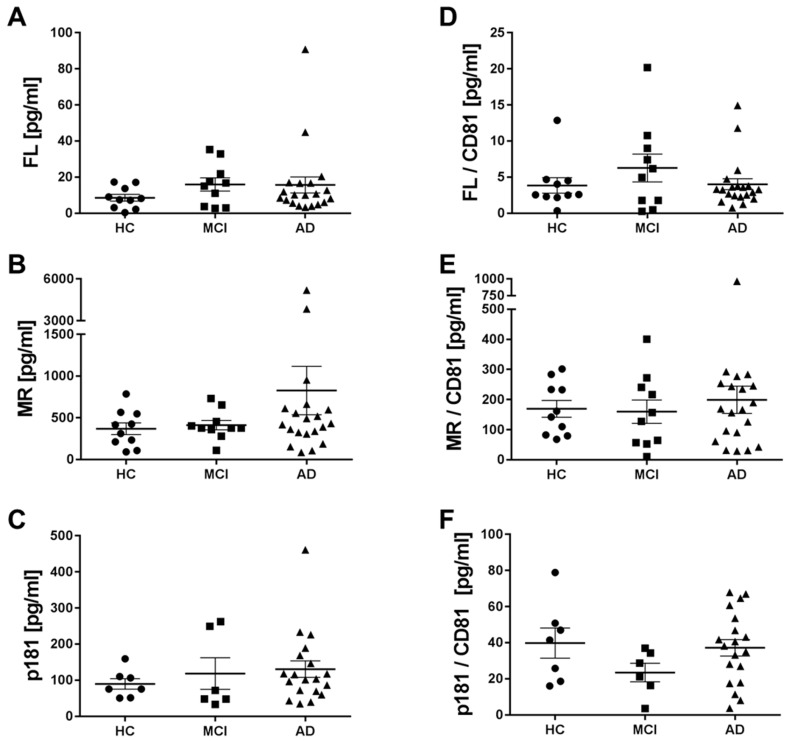
Levels of different types of tau in neuronal exosomes from healthy controls, AD and MCI subjects. (**A**–**C**) When full-length (FL), mid-region (MR), and ptau-181 (p181) results were stratified by clinical status, there was no apparent difference in the levels of tau in AD versus mild cognitively impaired (MCI), versus healthy controls (HC). (**D**–**F**) We also investigated if normalization based on the exosome marker protein CD81 could reveal a difference in relative tau levels in neuronal exosomes from the three diagnostic groups. Values were normalized by setting the sample with the lowest CD81 value to 1, while the rest of the samples were given relative CD81 concentrations, and tau values divided by the respective CD81 normalized value. Using one-way ANOVA, no group-specific differences were evident.

Two prior reports indicated that the levels of p181-tau in blood-derived neuronal exosomes were elevated in mild cognitive impairment (MCI) and AD relative to healthy control subjects [34,35] while in the one study to look, tau measured using a mid-region ELISA was not significantly elevated [34]. To explore whether p181-tau measurements and/or tau measured using our FL and mid-region assays could discriminate between AD and healthy controls (HC), we divided (post hoc) the 40 samples analyzed in our study into three diagnostic groups (HC, MCI, and AD) and compared the concentrations of tau analytes across the groups. There were no statistical differences observed between groups for any of the three tau analytes measured (Figure 7A–C).

Since the efficiency of exosome isolation could vary between samples and this might mask the differences in the amount of tau measured, we normalized measurements of tau in each sample relative to that of the established exosomal marker CD81 (Figure 7D–F). To ensure the precision of the CD81 measurements, we analyzed all 40 samples for CD81 on two independent days and the CD81 measurements on Day 1 and Day 2 were highly similar (Table 2). Two samples that gave values above the upper limit of detection on Day 1 yielded measurable values when further diluted on Day 2 (Figure S6). Although, there was considerable variation in the levels of CD81 across samples, normalizing tau results based on CD81 did not reveal any group differences in terms of tau analytes (Figure 7D–F). Other methods of normalization were also investigated, but none yielded a significant difference between the diagnostic groups (data not shown), and covariate or rank-sum analyses were not considered due to the small samples size. Thus, while mid-region, FL and p181 tau were detected in all of the samples examined, we could discern no disease-related changes in this small cohort.

## 3. Discussion

Until relatively recently, tau was considered to be an exclusively intra-neuronal protein, and the presence of tau in human CSF was thought to be a product of axonal damage or neuronal death [59]. It was also widely assumed that tau detected in CSF by mid-region directed ELISAs was intact, full-length protein. However, recent data suggest that: (i) tau is not purely intracellular [13,32,33,60,61,62,63,64,65,66,67,68,69], and (ii) the majority of extracellular tau is heavily truncated [13,39,40,41,60]. While most tau detected in the medium conditioned by neurons [13,60] or in the CSF of mammals is not encapsulated, but free-floating [39,40,41,69], several studies have reported detection of tau in extracellular vesicles (EVs) [13,32,33,57,61,64,65,67,68,70]. Most of the prior studies investigating tau in EVs utilized cell lines over-expressing exogenous tau [61,62,63,64,65,67,68,69,70] or used primary rodent cultures [13,32,33,66]. Here, we focused on tau in exosomes from human iPSC-derived neurons, human CSF, and human plasma.

Since exosomes have been suggested as potential vehicles by which small tau aggregates may be passed from neuron-to-neuron and the MTBR domains of tau are required for aggregation, we developed sensitive immunoassays to search for full-length (FL) tau both free-floating in solution and contained within exosomes. Our experiments are the first to unambiguously demonstrate that a small fraction (~1%) of extracellular tau in iN CM and human CSF is full-length. A number of studies using Western blotting have claimed to detect FL tau in human CSF and in CM from untransfected cells [33,71,72,73]. However, the limited sensitivity of Western blotting and the amount of tau measured by more sensitive means undermine the veracity of these claims. Specifically, numerous studies using validated mid-region assays have reported that the levels of mid-region ELISA-detected tau in healthy individuals is in the order of 100–400 pg/mL [47,74,75], values consistent with our analysis of the six healthy controls in this study. Assuming that the minimum signal detectable for a single protein band by Western blotting using chemiluminescent detection systems is ~100 pg/mL, that would necessitate loading ~0.3–1.0 mL of CSF to allow for detection. If there are multiple forms of tau in CSF, then the 100–400 pg/mL detected by ELISA would be spread over a number of bands, necessitating that even larger volumes of CSF be used. Given the small volumes of CSF that is used in most Western blotting studies and the abundance of albumin and IgG [76,77] (both of which migrate close to tau), and their notorious cross-reactivity with a range of antibodies, it is likely that bands attributed heretofor FL tau were artifacts.

The few studies that concentrated large volumes of CSF prior to Western blotting have each detected fragments of tau in the range of ~35–40 kDa, but did not detect FL tau [39,40,78]. Those results correspond well with the relative levels of tau we detected here in CSF by mid-region ELISA and FL Simoa, i.e., FL tau accounts for only ~1% of tau detected by mid-region assays. Although this current report presents data from only 2 CSF pools, in a separate study we readily detected low levels of FL tau in 70 individual CSF specimens. In that cohort, the FL signal typically accounted for ~1% and never more than 2% of the mid-region signal [79].

While the levels of free-floating tau are much higher than those found in EVs, FL tau contributed more of the mid-region signal inside exosomes than was the case for extra-vesicular tau. This relative enrichment of FL tau in exosomes was detected in CSF and plasma exosomes, and, for yet unknown reasons, was particularly strong in iN exosomes. At least a portion of tau is degraded by autophagy [80], and higher levels of tau are found in exosomes when tau is ectopically expressed at high levels [68], suggesting that when tau escapes autophagy, a process tightly linked to the site of exosomal genesis (multivesicular bodies), more tau is secreted in exosomes. Under normal circumstances, one would expect that only a small amount of tau would escape degradation and enter exosomes, and that the resulting material would, as suggested by our findings, tend to be full-length. In accord with our detection of FL tau in CSF exosomes, a recent study utilizing Western blotting also claimed to detect FL tau in CSF exosomes. However, there were neither positive controls to indicate the sensitivity of the detection system, nor negative controls to validate the bands attributed as tau [33]. Future studies should also investigate post-translation modifications (particularly phosphorylation and acetylation) on tau inside EVs, as this might give insight into the process(es) by which tau is directed to EVs. From a mechanistic stand point, analysis of tau in CSF exosomes is clearly important, but from a diagnostic view, the measurement of tau in exosomes is not particularly attractive since there already exists excellent CSF AD biomarkers that require smaller volumes of CSF [47,81,82].

In contrast, there is an urgent need for blood-based biomarkers that can replace or supplement current CSF markers. Given the demonstrated utility of quantifying tau in CSF, there is much interest in measuring tau in blood, but, unlike CSF, the contents of blood are influenced by many organs. Therefore, levels of blood tau may not be sensitive to minor pathobiological changes in brain. On the other hand, measurement of tau in brain-derived, blood-borne EVs should better reflect alterations occurring in brain. Recent evidence suggests that it is possible to isolate neuronal exosomes from blood plasma and that measurement of certain forms of tau in neuronal exosomes can be used as diagnostic and prognostic biomarkers [34,35]. However, in the current study, we could discern no disease specific change in the 3 tau analytes measured, one of which (p181 tau) was found to be elevated in AD in two prior studies [34,35]. Clearly, our results are concerning in terms of the utility of exosomal tau as a useful biomarker for AD. However, the small size of the cohort examined here and the fact that disease designation was based solely on clinical criteria now necessitate a study with a larger number of subjects who preferably have disease designation validated, either by autopsy or by measurements of accepted CSF AD biomarkers, i.e., Aβ42, mid-region tau, and p181 tau [47,81,82]. Similarly, it will be important to determine if the optimization steps utilized in our study had some unintended effects on recovery of exosomal tau. Nonetheless, our detection of small amounts of full-length, aggregation competent tau in blood neurally-derived exosomes is consistent with our detection of small amounts of FL tau in CSF and iN exosomes. However, it is not clear why more tau is recovered in neuronal plasma exosomes than in CSF exosomes. One possibility is that the ExoQuick method that is used to isolate exosomes from plasma is more effective or captures a more diverse range of EVs than the centrifugation protocol used to harvest exosomes from CSF. In this regard, others have reported that differential centrifugation approaches, such as those we used to isolate EV’s from CSF, are rather low yield and may not be highly pure [83]. Regarding different types of EVs, it is worth noting that certain cell culture experiments have detected more tau in MVs than exosomes [32]. Furthermore, since our assays do not discriminate between neuronal and non-neuronal (e.g. “big tau”) forms of tau, it is also possible that peripheral neurons and other peripheral cells may contribute to tau in the ExoQuick/L1CAM isolation method.

Regardless of the precise forms and sources of tau detected by the ExoQuick/L1CAM isolation method, a future question to address will be whether such exosomes contain aggregated tau capable of seeding monomeric tau in recipient cells. A corollary of such a finding might be the possibility of transmitting aggregation-competent tau by using blood products and would necessitate testing the infectious potential of exosomes containing tau aggregates. Similarly, it will also be important to examine if other diseases linked to altered tau metabolism exhibit changes in the amount and/or forms of tau found in neuronal exosomes.

## 4. Materials and Methods

### 4.1. Generation of Induced Neurons (Ins) from Induced Pluripotent Cells (Ipscs)

The YZ1 iPSC line [43] was used to prepare neurogenin 2 (Ngn2)-induced human neurons essentially, as described previously [45], and Figure 2A. iPSCs were maintained in medium containing DMEM/F12, Knockout Serum Replacement, penicillin/streptomycin, L-glutamine, MEM-NEAA, and β-mercaptoethanol (all from Invitrogen, Grand Island, NY, USA) with addition of 10 μg/mL basic fibroblast growth factor (bFGF; Millipore, Temecula, CA, USA) prior to media application. Neuronal differentiation was performed via a doxycycline-inducible neurogenin 2 system ([44] and Figure 2A). iPSCs were plated at a density of 95,000 cells/cm^2^ for viral infection. Ultrapure lentiviral titers were obtained from Alstem (Richmond, CA, USA) and used at the following concentrations: Tet-O-Ngn2-puro: 0.1 µL/50,000 cells; Tet-O-FUW-EGFP: 0.05 µL/50,000 cells; FUdeltaGW-rtTA: 0.11 µL/50,000 cells. To induce neurogenin 2 expression, doxycycline was added on iN day 1 (D1) (Figure 2A) at a concentration of 2 µg/mL. On iN D2, puromycin was added at 10 µg/mL and was maintained in medium thereafter. On iN D4, cells were plated on Matrigel (Corning, Bedford, MA, USA)-coated 96-well plates (150 cells/mm^2^) or 10 cm dishes (300–50 cells/mm^2^) and maintained in media consisting of Neurobasal, Glutamax, 20% dextrose, MEM-NEAA, B27 (all Gibco, Grand Island, NY, USA) and 10 ng/mL BDNF, CNTF, GDNF (PeproTech, Rocky Hill, NJ, USA). Half of the culture media was replaced every four days with fresh complete media. On iN D21, cells were characterized using live cell imaging to monitor neurite development (Figure 2B) and used for immunocytochemistry to assess expression of neuronal markers (Figure 2C–E).

### 4.2. Characterization of Induced Neurons (Ins) by Live Cell Imaging and Immunocytochemistry

On iN D21, iNs plated at 150 cells/mm^2^ in opaque-wall 96 well plates (Cat. #655090; Greiner, Monroe, NC, USA) were either live-imaged or fixed and stained for mature neuron-specific markers. Briefly, cells were washed extensively with filtered, warm artificial cerebrospinal fluid (aCSF; 124 mM NaCl, 2.8 mM KCl, 1.25 mM NaH_2_PO_4_, 26 mM NaHCO_3_), and live-imaged in 50 µL using brightfield and FITC filters with an IN Cell Analyzer 2200 (GE Healthcare, Milwaukee, WI, USA; 20 × 0.75 NA objective lens). Thereafter, adjacent wells were fixed with cold 4% paraformaldehyde/4% sucrose in PBS for 10 min at 4 °C, washed twice with PBS, quenched in 0.1 M glycine/PBS for 5 min, washed 4× with PBS, and transferred to 4 °C overnight. The following day, plates were warmed to room temperature for 30 min prior to immunostaining to minimize MatriGel interference. Cells were permeabilized for 5 min with 0.5% Triton-X 100, washed 3× with PBS, blocked in 3% BSA/PBS for 1 h, washed 3x and immunostained for MAP2 (1:2,000; Cat. #AB15452; MilliporeSigma, Billerica, MA, USA), β-III-tubulin (clone D71G9; 1:4000; Cat. #5568S; Cell Signaling, Danvers, MA, USA) or tau (clone Tau5; 1:500; Cat. #806402; BioLegend, Dedham, MA, USA) for 2 h in 3%BSA/PBST. Thereafter, cells were washed 6× with PBST and incubated with Alexa647 conjugated donkey-anti chicken (MAP2), rabbit (β-III-tubulin) or mouse (tau) secondary antibodies (all 1:500) and Alexa488 conjugated phalloidin (1:500; Cat. #A12379; ThermoFisher, Waltham, MA) for 1 h in 3%BSA/PBST. Plates were then washed 3× with PBST, incubated with DAPI (1:1000 in PBS) for 5 min, washed 3× with PBST, 2× with PBS and imaged using DAPI, FITC and Cy5 filters with an IN Cell Analyzer 2200 (20× 0.75 NA objective lens). Images were extracted and analyzed with IN Carta (GE Healthcare).

### 4.3. Isolation of Exosomes from in Conditioned Media (CM)

Media was collected on iN D23-33 from cells plated on 10 cm dishes (300–350 cells/mm^2^), pooled and then processed in 30 mL batches as described in Figure 3A. First, CM was centrifuged at 300× *g* and 4 °C for 10 min to pellet floating cells. Then, the upper 97% was recovered (supernantant 1; S1) and the remaining S1 was centrifuged at 2000× *g* and 4 °C for 10 min to pellet apoptotic bodies. Next, the upper 97% of this was recovered (S2) and stored at −80 °C. When required, S2 CM was thawed, pooled, and further diluted in NBM to a final volume of 30 mL/experiment. Diluted S2 was centrifuged at 10,000× *g* and 4 °C for 30 min to pellet microvesicles and cell debris. Finally, the upper 97% was recovered (S3) and centrifuged at 100,000× *g* and 4 °C for 70 min to pellet exosomes. A portion (~0.25 mL) of each fraction was stored at −80 °C. Finally, the upper 97% of S4 was removed and stored at −80 °C. The 100,000 g exosome pellet was washed in 1 mL DPBS (Millipore-Sigma, St. Louis, MO, USA) containing protease and phosphatase inhibitors (ThermoFisher, Carlsbad, CA, USA), and re-pelleted at 100,000 g for 70 min. Exosomes were lysed in 75 µL M-PER (Cat. #78501; ThermoFisher, Carlsbad, CA, USA) containing 1× phosphatase and protease inhibitors and subjected to two rounds of freezing on dry ice and thawing in a room temperature water bath. All of the dilution and concentration steps were considered when calculating tau concentrations.

### 4.4. Western Blotting Analysis of in Cells and Exosomes

Samples were electrophoresed on pre-cast 4–12% polyacrylamide bis-tris gels using MOPS running buffer (ThermoFisher, Waltham, MA, USA) and transferred onto 0.2 μM nitrocellulose membranes at 400 mA for 2 h. Thereafter, blots were blocked in Odyssey blocking buffer (Li-COR, Lincoln, NE, USA) and membranes cut to allow the analysis of the same samples for Alix (clone 3A9, 1 μg/mL; Cell Signaling, Danvers, MA, USA), Tsg101 (clone EPR7171(B), 1 μg/mL; Abcam, Cambridge, MA, USA), Flotillin-1 (clone 18, 1 μg/mL; BD Biosciences, San Jose, CA), PrP (clone ICSM-18, 1 μg/mL; D-Gen, London, England), and TOM20 (clone 29, 0.25 µg/mL; BD Biosciences, San Jose, CA, USA). Membranes were incubated overnight at 4 °C in primary antibody solution, followed by 6 × 10 min washes with Tris buffered saline with 0.05% Tween-20 (TBST). Then, membranes were incubated with infrared-labeled goat anti-mouse 800 or donkey-anti-rabbit 680 secondary antibodies (Li-COR, Lincoln, NE, USA) for 1 h at room temperature, followed by 6 × 10 min washes with TBST. Immunoreactive bands were visualized using a Li-COR Odyssey CLx infrared imaging system (Li-COR, Lincoln, NE, USA).

### 4.5. Mid-Region Tau ELISA

This assay was performed essentially, as described previously ([13] and Figure 1C). Half-area, high binding 96-well plates (Cat. #675077; Greiner, Monroe, NC, USA) were coated with 2.5 μg/well BT2 (Cat. #MN1010; ThermoFisher, Carlsbad, CA, USA) diluted in TBS (pH 7.4) for 1 h at 37 °C with shaking (300 rpm). Plates were then washed three times with 100 µL TBST prior to blocking in 100 µL TBS containing 3% BSA for 2 h at room temperature and 300 rpm. Plates were washed 3 times with 100 µL TBST before 25 µl samples and standards (diluted in glycine/MPER buffer) were applied in triplicate and agitated for 16 h at 4 °C. The following day, 25 µL alkaline phosphatase conjugated Tau5 diluted 1:250 in TBST containing 1% BSA was added directly to the plates without washing and incubated for 1 h at room temperature and 300 rpm. Finally, plates were washed five times with 100 µL TBST before 50 µL Tropix Sapphire II (Cat. #T2214; ThermoFisher, Carlsbad, CA, USA) detection reagent was added and incubated for 30 min at room temperature and 300 rpm. Fluorescence was measured in a CLARIOstar plate reader (BMG Labtech, Cary, NC, USA) and standard curves were fitted to a five-parameter logistic function with 1/Y^2^ weighting using MasterPlex ReaderFit (MiraiBio, Alameda, CA, USA). Lower limit of quantitation (LLoQ) was determined by calculating the average + 9 standard errors and 100 ± 20% recovery for each standard. For the experiments shown, the LLoQ of the mid-region assay was 7.81 pg/mL.

### 4.6. Full-Length Tau ELISA

This assay was performed as with the mid-region assay except different capture and detection antibodies were used. Here, Tau12 (Millipore, Billerica, MA, USA) was used for capture and TauAB (a generous gift from Andy Billinton and Mike Perkinton of MedImmune) for detection (Figure 1B). For the experiments, shown the LLoQ of the FL assay was 16.35 pg/mL.

### 4.7. Isolation of Exosomes from Human Cerebrospinal Fluid (CSF)

All of the samples were obtained and used under IRB approval (Walsh 2016P0002G1/BWH). Individual CSF samples were from six healthy female volunteers participating in the Harvard Biomarker Study (HBS) and CSF pools were obtained from Crimson Clinical Discards Partners (CCDP) Biobank. CSF pool A was the product of CSF collected from four individuals and pool B from three subjects. Under the terms of the IRB, demographic information is available for the samples obtained from either the CCDP biobank or the HBS. For the six HBS samples, 10 mL aliquots of CSF were centrifuged exactly as described for iN CM in Figure 3A, and CSF pools A, and B were processed in an identical manner. All of the CSF samples were free of blood and cellular contaminants.

### 4.8. Plasma Samples and Isolation of Neuronal Exosomes

Forty, 500 µL aliquots of plasma were obtained from the Harvard NeuroDiscovery Center Biomarker Study. Samples came from donors free of cognitive impairment (CN, *n* = 10), donors with mild cognitive impairment (MCI, *n* = 10), and with mild or moderate AD (*n* = 20). Demographic and clinical data are provided in Table 1. Importantly, experimenters were blind to the disease designation of donor samples.

Neuron-derived exosomes were isolated from plasma using a slightly modified version of the procedure pioneered by the Goetzl/Kapogiannis group [34]. Five µL thrombin (Cat. #TMEXO-1; System Bioscience, Palo Alto, CA, USA) was added to 500 µL aliquots of plasma to induce clot formation and allow the removal of fibrin and related proteins. Reactions were mixed by inversion and incubated for 30 min at room temperature before dilution with 495 µL Ca^2+^- and Mg^2+^-free Dulbecco’s Phosphate-Buffered Saline (DPBS) (Sigma-Aldrich, St. Louis, MO, USA) containing 3x phosphatase (Cat. #78426; ThermoFisher, Carlsbad, CA, USA) and protease (Cat. #11697498001; Roche, Branchburg, NJ, USA) inhibitors. Thereafter, the samples were centrifuged at 6000× *g* for 20 min at 4 °C and the supernatant transferred to a new tube. Next, 252 µL ExoQuick (Cat. #EXOQ20A-1, System Bioscience, Palo Alto, CA, USA) was added to the supernatants, samples mixed by inversion and left to stand at 4 °C for 1 h. Vesicles present in the serum were recovered by centrifugation at 1500× *g* for 20 min at 4 °C and resuspended by vortexing in 500 µL MilliQ water containing 3× phosphatase and protease inhibitors. To ensure the complete resuspension of the ExoQuick pellets, samples were mixed overnight at 4 °C on a vertical rotating mixer.

To isolate neuronal exosomes from the heterogenous suspensions, 4 µg biotinylated anti-CD171/L1CAM antibody (Cat. #13-1719-82; eBioscience, San Diego, CA, USA) in 42 µL 3% Bovine Serum Albumin in DPBS (BSA/DPBS) was added to the resuspended vesicles and incubated for one hour at 4 °C with rotation. Thereafter, 15 µL of pre-washed Streptavidin-Plus UltraLink Resin (Cat. #53116, ThermoFisher, Danvers, MA, USA) in 25 µL 3% BSA/DPBS was added to each sample and incubated for 4 h at 4 °C with rotation. Neuronal exosomes bound to the antibody/resin complex were recovered by centrifugation at 200× *g* for 10 min at 4 °C and washed once with 3% BSA/DPBS^-/-^ before elution in 200 µL 0.1 M glycine (pH 3.0). Resin was removed by centrifugation at 4500× *g* for 5 min at 4 °C and 5 µL of the neuronal exosome-containing supernatant was diluted in 995 µL filtered DPBS for NanoSight analysis. The remaining 195 µL supernatant was neutralized with 15 µL of 1 M TrisHCl (pH 8.0) and exosomes were lysed by adding 360 µL M-PER (Cat. #78501; ThermoFisher, Carlsbad, CA, USA), 25 µL 3% BSA/DPBS containing 1× phosphatase and protease inhibitors, and 2 freeze-thaw cycles before downstream analysis.

### 4.9. CD81 ELISA

The ELISA kit from Cusabio (Cat. #CSB-EL004960HU; Cusabio, College Park, MD, USA) was used to measure CD81. Briefly, 100 μL of sample or standard prepared in glycine/M-PER buffer (40 mM glycine pH 3.0, 0.1% BSA/DPBS, 30 mM TrisHCl pH 8.0, 52% M-PER) was added to pre-coated wells and incubated for 2 h at 37 °C with shaking at 300 rpm. Thereafter, 100 μL of biotinylated secondary antibody was added and incubated for 1 h at 37 °C with shaking at 300 rpm. Wells were washed three times before addition of 100 μL HRP-avidin per well. Plates were incubated for 1 h at 37 °C with shaking at 300 rpm, and then washed five times before 90 μL TMB substrate was added and incubated at 37 °C. Plates were developed for 15 min, stopped by the addition of 50 μL stop solution, and absorbance measured at 450 nm in a CLARIOstar plate reader (BMG Labtech, Cary, NC, USA). For the experiments shown, the LLoQ was between 0.31 and 1.25 ng/mL.

### 4.10. Phosphorylated Threonine-181 (p181) Tau ELISA

Tau phosphorylated at threonine 181 was measured using the Innotest ELISA (Cat. #80317; Fujirebio, Malvern, PA). Briefly, 75 μL sample (diluted 1:2 in assay buffer) or standards was added to each well together with 25 μL of Conjugate Working Solution 1 provided by the manufacturer. Plates were agitated overnight at 4 °C and 300 rpm. After 5 washes, 100 μL Conjugate Working Solution 2 was added to each well and plates were incubated for 1 h at 25 °C. Next, wells were washed five times and 100 μL of Substrate Working Solution was added to each well and left for 30 min at 25 °C in the dark. Thereafter, 50 μL of Stop Solution was added to each well and absorbance determined at 450 nm. Standard curves were fitted and LLoQs determined as with the mid-region assay. CSF samples provided by the manufacturer were added as internal controls and used to monitor plate-to-plate variability. Values for the internal controls were within the defined concentration range and the plate-to-plate variance was low (70.2 and 83.4 pg/mL). For the experiments shown, the LLoQ was 24.5 pg/mL.

### 4.11. Full-Length Tau Simoa-Based Assay

For sample types in which the levels of FL tau were too low to measure by ELISA (i.e., CSF and plasma exosome lysates), tau was measured using a Simoa (single molecule analysis)-based version of our FL assay and a Simoa HD-1 platform (Quanterix, Lexington, MA, USA). The C-terminal antibody, TauAB (a generous gift from Andy Billinton and Mike Perkinton of MedImmune), was used for capture and the N-terminal Tau12-biotin antibody (Cat. #MAB2241; Millipore, Billerica, MA, USA) for detection. Beads were activated with 1-Ethyl-3-(3-dimethylaminopropyl)carbodiimide (EDC; Cat. #22980; ThermoFisher, Waltham, MA, USA) and coated with TauAB. Tau12 was biotinylated using N-hydroxysuccinimide-polyethylene glycol (NHS-PEG4)-biotin (Cat. #21330; ThermoFisher, Waltham, MA, USA). Standards (human tau 381; Cat. #T0201; MilliporeSigma, St. Louis, MO, USA) and blanks were prepared using sample buffer and assayed in triplicates while samples were assayed in duplicate. Standard curves were fitted and LLoQs determined as with the mid-region and p181 tau assays. For the experiments shown, the LLoQ of the FL Simoa assay was 0.74 pg/mL. Full validation of this assay will be described in a separate publication [79].

### 4.12. Tsg101 MSD Immunoassay

To enable the measurement of Tsg101 in exosome lysates, we developed an in-house Meso Scale Discovery (MSD)-based assay. The wells of MSD GOLD 96-well streptavidin plates (Cat. #L15SA-1; MSD, Stoughton, MA, USA) were blocked with 150 µL of 5% Blocker A (Cat. #R93BA-4; MSD) in TBST for 1 h at room temperature and gentle agitation on an orbital shaker (300 rpm). Plates were washed three times with 1× Tris wash buffer (Cat. #R61TX-1; MSD) and 25 µL/well of biotinylated mouse anti-Tsg101 (clone 4A10—2 µg/mL) capture antibody (Cat. #NB200-112B; Novus, Littleton, CO, USA) was added in 1% Blocker A/TBST. Coating was carried out for 1 h at room temperature with shaking at 300 rpm, and plates were washed three times with 1× Tris wash buffer before adding 25 µL/well standards or samples in triplicate. Standards were prepared by diluting recombinant Tsg101 protein (Cat. #H00007251-P01; Novus, San Diego, CA, USA) in glycine/M-PER buffer: 400,000 ng/mL, 100,000 ng/mL, 40,000 ng/mL, 20,000 ng/mL, 10,000 ng/mL, 5000 ng/mL, and 2500 ng/mL. After incubation for 1 h at room and gentle agitation (300 rpm), plates were washed three times with wash buffer and incubated for 1 h at room temperature and 300 rpm with rabbit SULFO-tag anti-Tsg101 detection antibody (1 µg/mL) (Cat. #ab133586; Abcam, Cambridge, MA, USA) diluted in 1% Blocker A/TBST. Plates were then washed 3× with wash buffer before addition of 1× Read Buffer (Cat. #R92TC-2; MSD) to allow for electrochemiluminesence detection (150 µL/well). A Sector imager was used to measure the intensity of emitted light, thus allowing the quantitative measurement of analytes present in the samples. The LLoQ for this assay, calculated as described for the other tau immunoassays, was between 2.5 and 5 ng/mL.

### 4.13. Characterization of Exosomes by Nanoparticle Tracking Analysis (NanoSight)

Blood-derived neuronal exosomes diluted 1:200 in filtered DPBS were injected into a NanoSight LM10 (Malvern Instruments, Westborough, MA, USA) to assess vesicle size and concentration. Each sample was measured in duplicate, with 5 × 1 min nanoparticle tracking analysis videos captured at a constant flow rate of 40 μL/min. Extracellular vesicles were detected at a camera gain of 10 and threshold value of 5. Resultant concentration and size distribution data were averaged within-sample using 10 measurements (i.e., two replicates with five analyses each). To determine global distribution of all vesicles measured, particle counts were binned into 5 nm intervals and each interval expressed as a percentage of total particles analyzed within a given sample.

## Figures and Tables

**Table 1:** Demographics and clinical characteristics of plasma donors.

	HC	MCI	Mild AD	Moderate AD
Subjects (n)	10	10	10	10
Age (Mean ± SD)	75.90 ± 8.67	76.00 ± 7.97	75.60 ± 12.89	75.00 ± 11.34
MMSE (Mean ± SD)	29.67 ± 0.52	28.10 ± 1.20	22.80 ± 6.18	15.00 ± 5.24
Sex:				
Female	7	5	4	7
Male	3	5	6	3
*APOE* allele:				
ε4/ε4	-	1	2	2
ε4/ε3	3	2	4	4
ε4/ε2	-	1	-	-
ε3/ε3	6	5	3	4
ε3/ε2	1	1	1	-

SD, standard deviation; HC, healthy control; MCI, mild cognitive impairment; AD, Alzheimer’s disease.

**Table 2:** Raw values for all assays ordered by diagnostic group.

Diagnosis	CDR	p181 tau (pg/mL)	MR tau (pg/mL)	FL tau (pg/mL)	CD81 A (ng/mL)	CD81 B (ng/mL)	CD81 Avg. (ng/mL)	Tsg101 (ng/mL)	Vesicles/mL (65–305 nm)
HC	0	106.32	91.31	3.09	1.85	2.53	2.19	4.24 *	7.39 × 10^10^
HC	0	23.27 *	212.09	9.02	3.09	3.19	3.14	6.28 *	9.30 × 10^10^
HC	0	110.09	564.53	17.14	12.07	10.21	11.14	25.34	6.91 × 10^10^
HC	0	24.01 *	106.49	17.30	1.89	2.48	2.18	17.03	7.75 × 10^10^
HC	0	51.65	785.36	7.19	3.81	5.20	4.50	62.21	1.20 × 10^11^
HC	0	25.21 *	310.33	0.34 *	1.38	1.97	1.67	8.47 *	5.88 × 10^10^
HC	0	75.76	415.53	7.46	3.94	5.59	4.77	13.52	4.90 × 10^10^
HC	0	75.47	425.05	8.22	2.49	3.43	2.96	12.83	9.93 × 10^10^
HC	0	159.50	547.89	13.67	4.81	6.23	5.52	26.26	8.64 × 10^10^
HC	0	50.81	232.69	2.18	1.19	2.06	1.63	10.45	1.12 × 10^11^
MCI	0.5	47.93	376.09	21.84	4.37	5.22	4.79	6.46 *	1.37 × 10^11^
MCI	0.5	71.93	109.38	3.74	2.98	3.83	3.41	23.66	5.14 × 10^10^
MCI	0.5	32.98	375.45	16.79	2.34	2.73	2.54	8.07 *	9.68 × 10^10^
MCI	0.5	22.35 *	279.21	11.02	2.42	3.37	2.90	7.85 *	7.86 × 10^10^
MCI	0.5	19.65 *	653.48	32.91	2.68	2.62	2.65	20.22	9.27 × 10^10^
MCI	0.5	19.29 *	455.32	15.03	2.11	3.33	2.72	5.88 *	6.73 × 10^10^
MCI	0.5	47.58	358.76	2.95	2.30	3.09	2.70	10.80	8.13 × 10^10^
MCI	0.5	249.49	730.90	17.56	^#^	113.64	113.64	617.83 ^#^	1.02 × 10^11^
MCI	0.5	262.32	402.86	35.30	31.68	19.31	11.54	106.61	1.37 × 10^11^
MCI	0.5	38.48 *	375.07	2.74	2.07	2.47	9.50	6.00	7.34 × 10^10^
AD	1	103.42	518.38	10.19	5.05	5.07	5.06	14.51	5.76 × 10^10^
AD	1	461.08	3850.19	90.77	^#^	200.64	200.64	3203.27 ^#^	9.62 × 10^10^
AD	1	69.33	390.15	3.32	2.23	3.17	2.70	8.99 *	5.49 × 10^10^
AD	0.5	101.36	952.47	10.04	5.50	6.71	6.11	113.91	8.26 × 10^10^
AD	2	115.47	360.99	4.80	2.39	3.79	3.09	13.05	5.97 × 10^10^
AD	2	233.30	152.63	16.61	7.11	9.09	8.10	30.39	8.01 × 10^10^
AD	1	117.66	105.67	5.62	2.47	3.16	2.82	6.32 *	5.30 × 10^10^
AD	1	168.64	186.16	10.73	6.80	7.56	7.18	83.26	7.15 × 10^10^
AD	1	188.85	337.34	16.71	4.71	6.75	5.73	20.00	1.21 × 10^10^
AD	0.5	59.97	417.40	3.88	2.34	3.23	2.78	32.55	8.29 × 10^10^
AD	2	34.95	490.67	7.25	2.36	4.13	3.25	11.66	6.81 × 10^10^
AD	2	146.63	663.16	8.15	3.10	4.27	3.69	16.34	1.18 × 10^11^
AD	2	118.26	83.97	8.48	3.86	5.60	4.73	8.62 *	1.39 × 10^11^
AD	0.5	43.28	596.59	44.91	6.58	5.80	6.19	42.78	1.09 × 10^11^
AD	2	39.38	612.44	6.02	8.32	7.53	7.92	29.48	8.97 × 10^10^
AD	2	86.36	555.09	11.91	2.71	3.80	3.26	11.00	1.14 × 10^11^
AD	1	19.47 *	322.82	16.99	1.51	2.19	1.85	7.52 *	8.48 × 10^10^
AD	2	96.47	5198.57	20.36	8.74	8.77	8.75	81.17	1.39 × 10^11^
AD	2	226.10	305.60	12.74	4.69	6.28	5.49	14.62	1.89 × 10^11^
AD	2	71.86	431.69	4.05	3.42	4.90	4.16	97.17	9.51 × 10^10^

HC, healthy control; MCI, mild cognitive impairment; AD, Alzheimer’s disease; CDR, clinical dementia rating; MR, mid-region; FL, full-length. CD81 ‘A’ and ‘B’ denote measurements of the same samples on different days. Values with * and ^#^ indicate measurements below the LLoQ and above ULoQ, respectively.

## Supplementary Materials

Supplementary materials can be found at http://www.mdpi.com/1422-0067/19/3/663/s1.

## Abbreviations

Aβ	Amyloid β-protein
aCSF	Artificial cerebrospinal fluid
AD	Alzheimer’s disease
Alix	Apoptosis-linked gene 2-interacting protein X
ANOVA	Analysis of variance
BDNF	Brain derived neurotrophic factor
CCDP	Crimson Clinical Discards Program
CD81	Cluster of differentiation 81
CM	Conditioned media
CNTF	Ciliary neurotrophic factor
CSF	Cerebrospinal fluid
DIV	Day in vitro
EV	Extracellular vesicle
FL	Full-length
GDNF	Glial derived neurotrophic factor
HBS	Harvard Biomarker Study
HC	Healthy control
IgG	Immunoglobulin G
iN	Induced neuron
iPSC	Induced pluripotent stem cell
L1CAM	Neural cell adhesion molecule L1
LLoQ	Lower limit of quantitation
mAb	Monoclonal antibody
MAP-2	Microtubule-associated protein 2
MCI	Mild cognitive impairment
MEM-NEAA	Minimum essential medium with non-essential amino acids
MSD	Meso Scale Discovery
MTBR	Microtubule binding region
MV	Microvesicle
MVB	Multivesicular body
p181	Tau phosphorylated at threonine 181
PrP	Prion protein
TBST	Tris buffered saline with 0.05% Tween-20
Tsg101	Tumor susceptibility gene 101
TOM20	Translocase of the outer membrane-20
ULoQ	Upper limit of quantitation
