# Supplementary material for: Detection of Aggregation-Competent Tau in Neuron-Derived Extracellular Vesicles
Source: Int J Mol Sci. 2018 Feb 27;19(3):663. doi: 10.3390/ijms19030663 (PMC5877524; doi:10.3390/ijms19030663)
Supplement: Supplementary file 1 [file ijms-19-00663-s001.pdf]

Supplemental Figure Legends

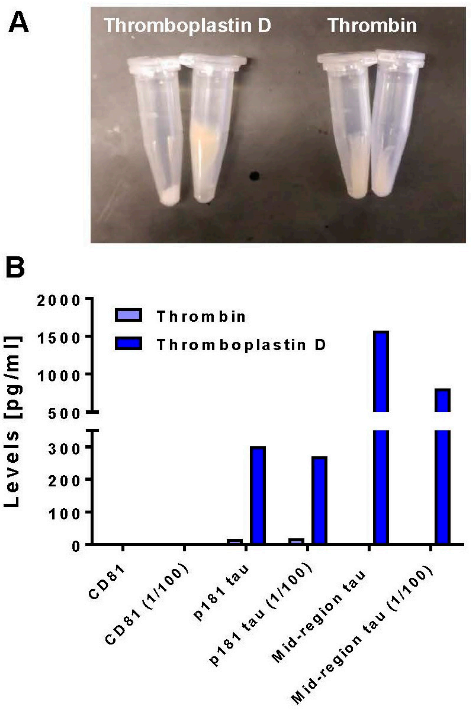

Figure S1. Comparison of thrombin versus thromboplastin D as a method to obtain serum from plasma. (**A**) Both factors induced the coagulation of plasma. However, thrombin tended to produce a more homogeneous clot size. (**B**) Thromboplastin D and thrombin, diluted in PBS to the concentration used in plasma samples, were analyzed for CD81, mid-region tau, and p181-tau. Neither thromboplastin D nor thrombin produced a CD81 signal. However, thromboplastin D yielded very high p181 and mid-region tau signals even when diluted 100-fold, whereas thrombin did not produce a significant signal in either assay.

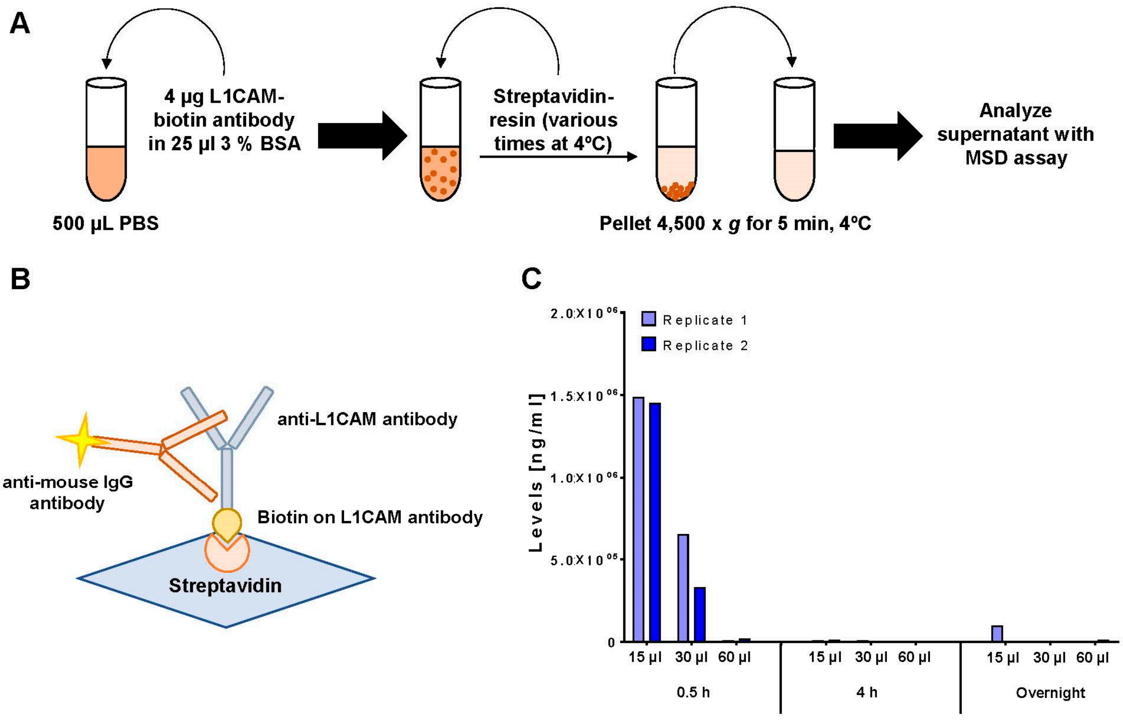

Figure S2. Increasing incubation time or the amount of streptavidin resin ensures maximal capture of biotinylated anti-L1CAM. (**A**) Schematic of the protocol used to determine the optimal concentration of streptavidin resin and incubation time to effectively capture biotinylated anti-L1CAM antibody. Four µg of L1CAM-biotin antibody in 500 µl of PBS was incubated with increasing amounts of streptavidin resin (15 µl, 30 µl and 60 µl) for increasing times (0.5 hour, 4 hours, or overnight). Streptavidin resin was pelleted and supernatants were tested for the presence of free biotinylated anti-L1CAM. (**B**) Streptavidin coated MSD plates were used to capture biotinylated L1CAM (blue IgG), and this was detected using a SULFO-TAG anti-mouse antibody (red IgG). (**C**) Comparison of the relative amounts of free-floating biotinylated anti-L1CAM antibody detected when different amounts of streptavidin resin and different incubation times were tested.

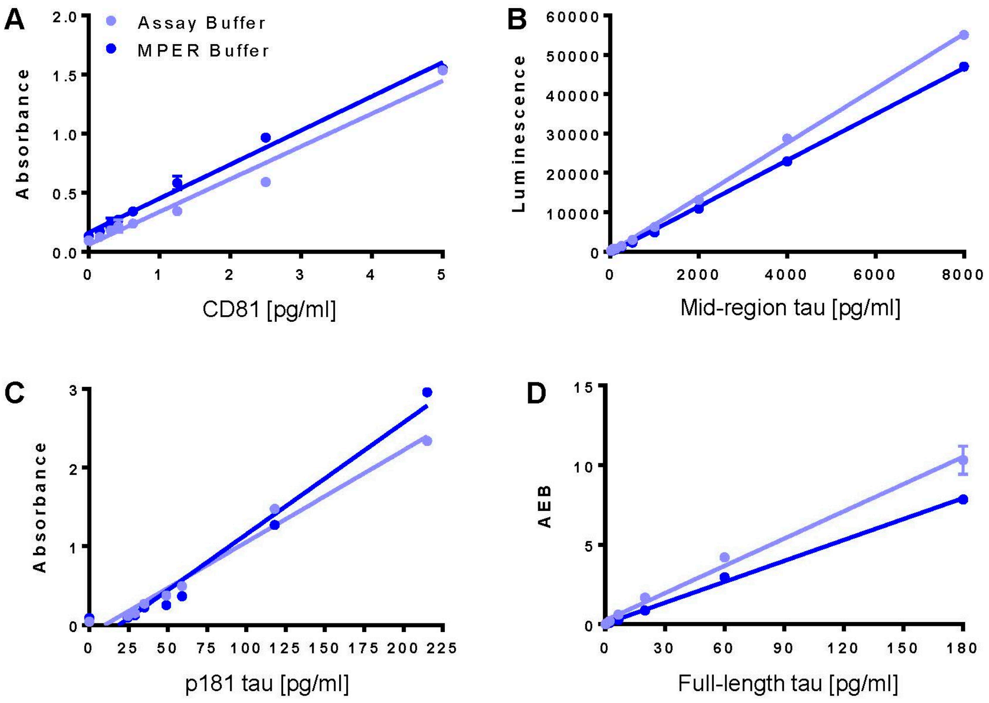

Figure S3. The exosome lysis buffer is compatible with immunoassays for (**A**) CD81, (**B**) mid-region tau, (**C**) p181-tau and (**D**) full-length tau. Standards were diluted in normal assay buffer or exosome lysis buffer and measured on the same plate for each assay. Curves are plots of raw output values (y-axis) the concentration of the respective proteins (x-axis).

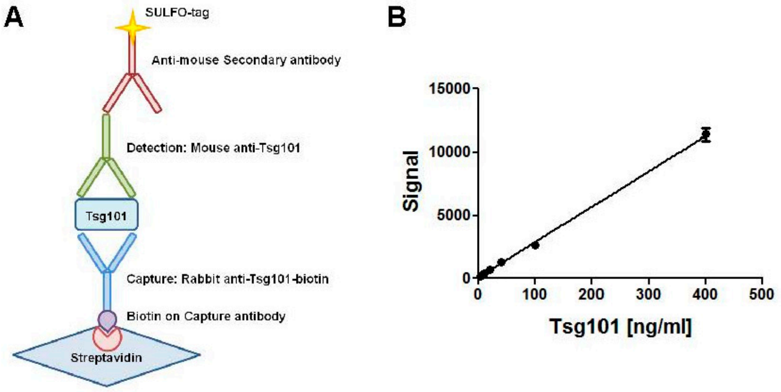

Figure S4. Design and linearity of the in-house Tsg101 MSD immunoassay. (**A**) Schematic depicting the Tsg101 MSD immunoassay. Briefly, biotinylated anti-Tsg101 antibody is adsorbed onto streptavidin-coated plates and used to capture Tsg101. Bound Tsg101 is then detected by a mouse anti-Tsg101 antibody and a SULFO-tagged anti-mouse secondary antibody. (**B**) Recombinant Tsg101 was diluted in exosome lysis buffer and detected as shown in panel (**A**).

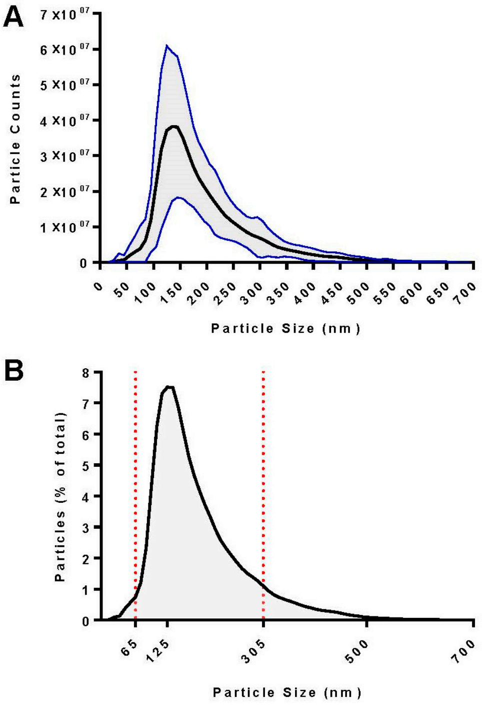

Figure S5. Particle distribution of blood-derived neuronal exosomes. Prior to lysis, 5 µl eluted exosome supernatant was diluted 200-fold in filtered DPBS and analyzed by NanoSight. (**A**) Size distribution of particle counts obtained from 40 plasma exosome samples based on two independent measurements, each with 5 replicates. Values represent the mean ± SD of 400 measurements. (**B**) Particle sizes were binned into 5 nm intervals with each interval expressed as a percentage of total particles analyzed within a given sample. Red dotted lines indicate that 90% of particles measured across 400 measurements fall between 65 and 305 nm.

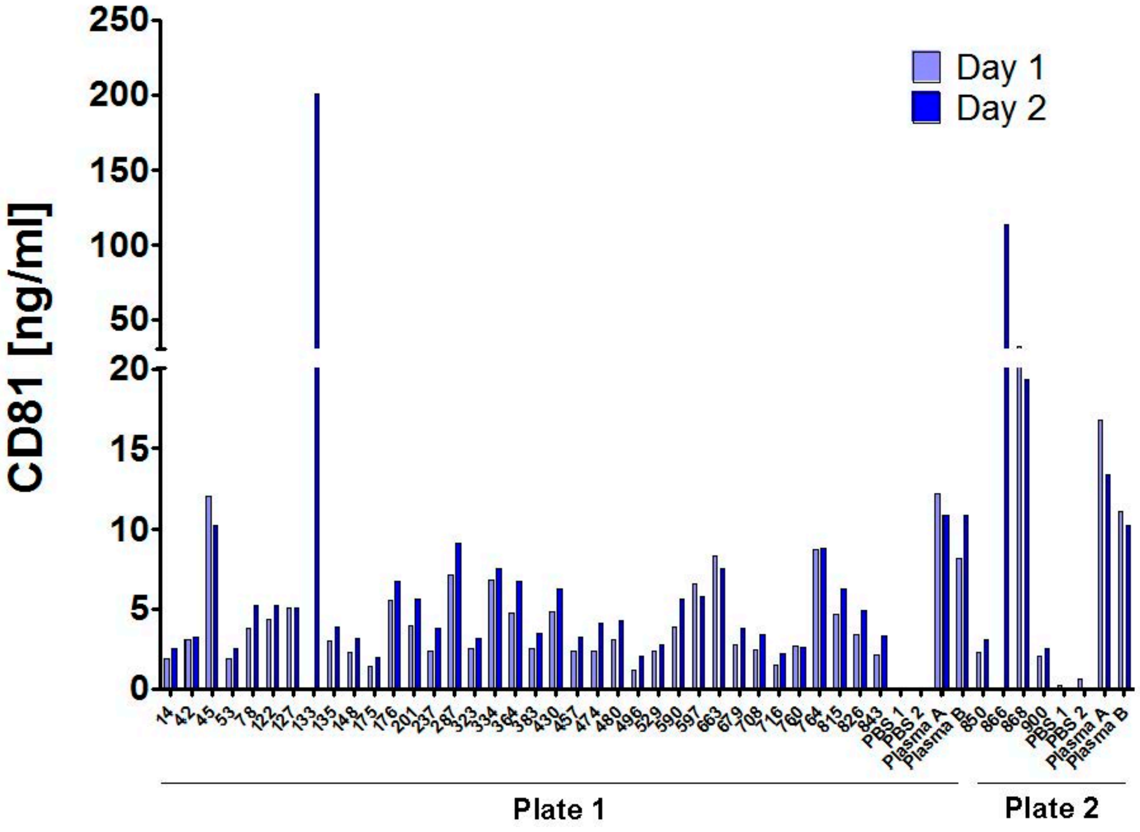

Figure S6. Quantification of CD81 levels in blood-derived neuronal exosome lysates from 40 donors. CD81 levels were determined on two different days; the first experiment (Day 1) was performed on undiluted samples and the second experiment (Day 2) was performed on samples diluted 1.8-fold. Samples denoted 'Plasma A' and 'Plasma B' served as internal plate controls.
